# ASP4058, a Novel Agonist for Sphingosine 1-Phosphate Receptors 1 and 5, Ameliorates Rodent Experimental Autoimmune Encephalomyelitis with a Favorable Safety Profile

**DOI:** 10.1371/journal.pone.0110819

**Published:** 2014-10-27

**Authors:** Rie Yamamoto, Youhei Okada, Jun Hirose, Tadatsura Koshika, Yuka Kawato, Masashi Maeda, Rika Saito, Kazuyuki Hattori, Hironori Harada, Yasuhisa Nagasaka, Tatsuaki Morokata

**Affiliations:** 1 Tsukuba Research Center, Drug Discovery Research, Astellas Pharma Inc., Ibaraki, Japan; 2 Kashima R&D Center, Drug Discovery Research, Astellas Pharma Inc., Osaka, Japan; University of Düsseldorf, Germany

## Abstract

Sphingosine-1-phosphate (S1P) is a biologically active sphingolipid that acts through the members of a family of five G protein-coupled receptors (S1P_1_–S1P_5_). S1P_1_ is a major regulator of lymphocyte trafficking, and fingolimod, whose active metabolite fingolimod phosphate acts as a nonselective S1P receptor agonist, exerts its immunomodulatory effect, at least in part, by regulating the lymphocyte trafficking by inducing down regulation of lymphocyte S1P_1_. Here, we detail the pharmacological profile of 5-{5-[3-(trifluoromethyl)-4-{[(2*S*)-1,1,1-trifluoropropan-2-yl]oxy}phenyl]-1,2,4-oxadiazol-3-yl}-1*H*-benzimidazole (ASP4058), a novel next-generation S1P receptor agonist selective for S1P_1_ and S1P_5_. ASP4058 preferentially activates S1P_1_ and S1P_5_ compared with S1P_2, 3, 4_ in GTPγS binding assays *in vitro*. Oral administration of ASP4058 reduced the number of peripheral lymphocytes and inhibited the development of experimental autoimmune encephalomyelitis (EAE) in Lewis rats. Further, ASP4058 prevented relapse of disease in a mouse model of relapsing-remitting EAE. Although these immunomodulatory effects were comparable to those of fingolimod, ASP4058 showed a wider safety margin than fingolimod for bradycardia and bronchoconstriction in rodents. These observations suggest that ASP4058 represents a new therapeutic option for treating multiple sclerosis that is safer than nonselective S1P receptor agonists such as fingolimod.

## Introduction

Multiple sclerosis (MS) is an idiopathic inflammatory disease of the central nervous system (CNS). Approximately 80% of patients present with relapsing-remitting disease that typically passes through phases of relapse with full recovery, relapse with persistent deficit, and secondary progression [1]. The disease is progressive from onset in 20% of patients and is therefore termed primary progressive [1]. Evidence indicates a central role for the immune system in the pathogenesis of MS, in which autoreactive lymphocytes infiltrate the CNS and attack myelin sheaths, leading to demyelination and axonal damage. Therefore, targeting the immune response is currently the main treatment for MS [2].

Sphingosine-1-phosphate (S1P) is a biologically active sphingolipid, which is involved in the regulation of various physiological functions as well as pathophysiological processes [3], [4]. Five cell-surface G protein-receptors (S1P_1_, S1P_2_, S1P_3_, S1P_4_, and S1P_5_) specifically bind S1P. While S1P_1_, S1P_2_, and S1P_3_ are widely expressed, S1P_4_ expression is restricted mainly to cells of the immune system, and expression of S1P_5_ is primarily detected in the white-matter tracts of the CNS [5]. S1P_1_, the predominant S1P receptor expressed on lymphocytes, is a major regulator of lymphocyte trafficking [6]. The concentration of S1P is relatively high in blood (approximately 1 µM) but extremely low (subnanomolar) in tissue interstitium [7], and an S1P concentration gradient promotes the egress of lymphocytes from secondary lymphoid tissue into the bloodstream [6].

Fingolimod is a nonselective S1P receptor agonist approved by the United States Food and Drug Administration in 2010 as the first oral treatment for relapsing forms of MS [8]. Sphingosine kinase phosphorylates fingolimod *in vivo*, which then acts as an agonist of four of the five S1P receptors (S1P_1_, S1P_3_, S1P_4_, S1P_5_) [9]. Fingolimod exerts its immunomodulatory effect, at least in part, by inducing internalization of S1P_1_ on lymphocytes, which reduces the responsiveness of these cells to the S1P gradient and inhibits egress of lymphocytes from secondary lymphoid tissue [10], [11]. In addition, fingolimod exerts direct effects on S1P receptors expressed on CNS cells, such as S1P_1_ on astrocytes and S1P_5_ on oligodendrocytes [12].

In clinical trials, fingolimod treatment was beneficial for patients with MS [13], [14], [15], and the annualized relapse rate in patients receiving fingolimod (0.20 and 0.16 in the 1.25 and 0.5 mg group, respectively) was significantly lower than in the patients receiving interferon β-1a (0.33), which is an established treatment for relapsing-remitting MS [13]. However, a pooled analysis of two phase 3 studies demonstrated that 6.1% of patients receiving 0.5 mg fingolimod and 11.0% of patients receiving 1.25 mg fingolimod experienced bradycardia (heart rate <50 beats per minute) after the first dose [16]. Heart rate reduction peaked at 4–5 h after dosing, and the mean heart rate decreased by 8 and 11 beats per minute at nadir with 0.5 and 1.25 mg, respectively [16]. Further, a reduction in the mean forced expiratory volume in 1 second (FEV1) was observed [13], [14]. The average reduction from baseline in the percentage of predicted FEV1 at month 6 was 8.8% and 2.8% in the patients receiving 5.0 mg and 1.25 mg fingolimod, respectively, as compared with 1.9% in the placebo group [14].

Preclinical data suggest that some of the adverse effects of fingolimod are caused by its interaction with S1P_3_ [17], [18]. We therefore assumed that S1P receptor agonists that do not engage S1P_3_ might provide a better therapeutic option for lymphocyte-mediated disease with fewer adverse effects than nonselective S1P receptor agonists such as fingolimod. To address this question, we developed ASP4058 as a novel S1P receptor agonist selective for S1P_1_ and S1P_5_. Here we present the *in vitro* profile of ASP4058, its *in vivo* effect on peripheral lymphocytes, and its efficacy in experimental autoimmune encephalomyelitis (EAE), which is a widely used animal model of MS. We also investigated the effect of ASP4058 on the heart rate and pulmonary function of rats compared with fingolimod.

## Materials and Methods

### Chemicals

**Figure 1:**
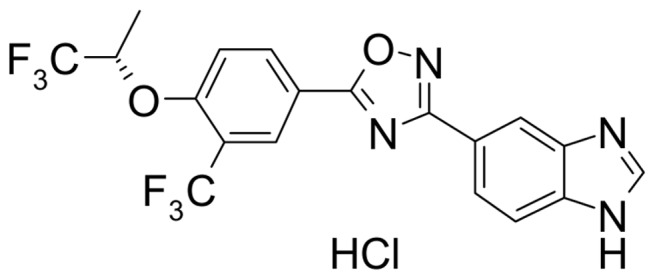
Chemical structure of ASP4058 (C19H12F6N4O2.HCl).

ASP4058 hydrochloride (5-{5-[3-(trifluoromethyl)-4-{[(2*S*)-1,1,1-trifluoropropan-2-yl]oxy}phenyl]-1,2,4-oxadiazol-3-yl}-1*H*-benzimidazole hydrochloride), ^14^C–labeled ASP4058 hydrochloride ([^14^C]ASP4058 hydrochloride), fingolimod hydrochloride (2-amino-2-[2-(4-octylphenyl)ethyl]propane-1,3-diol hydrochloride) and fingolimod phosphate (fingolimod-P) were synthesized at Astellas Pharma Inc. The structure of ASP4058 hydrochloride is shown in Fig. 1. Because fingolimod is a prodrug that requires phosphorylation to be an active metabolite, fingolimod-P was used in *in vitro* studies and *in vivo* studies in which compounds were administered intravenously. All dosages and concentrations of ASP4058, fingolimod, and fingolimod-P are expressed as their respective free-base equivalent.

### Ethics Statement

All animals were used in accordance with the guidelines of the Committee for Animal Experiments of Astellas Pharma Inc. All animal experimental procedures were approved by the Institutional Animal Care and Use Committee of Astellas Pharma Inc. Further, Astellas Pharma Inc., Tsukuba Research Center and Kashima Facilities are accredited by AAALAC International.

### Animals

Male and female Lewis rats, male Sprague Dawley rats and female SJL/J mice were purchased from Charles River Laboratories Japan, Inc. (Yokohama, Japan). Animals were maintained under a 12-h light-dark cycle and had free access to food and water except for in the analyses of the distribution of ASP4058 in the brain. For the latter experiments, rats were fasted with free access to water for approximately 16 h before ASP4058 administration, and feeding was resumed 4 h later. Surgery was performed on rats anesthetized with pentobarbital sodium or isoflurane inhalation, and all efforts were made to minimize suffering.

### Cell Lines

Chinese hamster ovary-K1 (CHO-K1) cells expressing human S1P_1_ (hS1P_1_), hS1P_2_, hS1P_3_, hS1P_4_, rat S1P_1_ (rS1P_1_), and rS1P_3_ were generated by transfecting CHO-K1 cells with pcDNA3.1 (+) vectors containing the full-length cDNA of each S1P receptor (Accession Numbers: hS1P_1_, NM_001400; hS1P_2_, NM_004230; hS1P_3_, NM_005226; hS1P_4_, NM_003775; rS1P_1_, NM_017301; rS1P_3_, NM_001271143). CHO-K1 cells expressing hS1P_5_ were generated by transfecting CHO-K1 cells with the pEF-BOS vector containing the full-length hS1P_5_ cDNA (Accession Number: NM_030760).

### GTPγS Binding Assay

Membranes were prepared from CHO-K1 cells expressing hS1P_1_, hS1P_2_, hS1P_3_, hS1P_4_, hS1P_5_, rS1P_1_, and rS1P_3_ based on the methods of Mandala [10] with modifications. Briefly, cells were washed with PBS, suspended in 1 mM Tris-HCl (pH 7.4), 0.1 mM EDTA, and 1× Complete protease inhibitor cocktail (Roche Diagnostics, Mannheim, Germany), and disrupted on ice using a dounce homogenizer. The homogenate was centrifuged for 10 min at 1000 *g* and the supernatant was centrifuged at 100,000 *g* for 60 min at 4°C. The pellet was suspended in 10 mM Tris-HCl (pH 7.4), 1 mM EDTA and stored at −80°C. ASP4058 and fingolimod-P were dissolved in dimethyl sulfoxide (DMSO) (Wako Pure Chemical Industries, Osaka, Japan) and then diluted with assay buffer (20 mM HEPES [pH 7.5], 100 mM NaCl, 10 mM MgCl_2_, 0.1% fatty acid-free bovine serum albumin, and 5 µM GDP) to various concentrations. Membranes (20 µg) were mixed with test-compound solution (final concentration, 1% [v/v] DMSO) and 50 pM [^35^S]-GTPγS (PerkinElmer, Waltham, MA, USA) in 150 µl of assay buffer. Membranes were incubated for 60 min at room temperature and collected onto GF/B filter plates (PerkinElmer), and then filter-bound radionuclides were measured using a TopCount NXT Microplate Scintillation and Luminescence Counter (PerkinElmer).

### Peripheral Lymphocyte Counts

Male Lewis rats were randomized by weight into each group and administered by gavage either a single or a once-daily dose for 21 days of ASP4058, fingolimod, or 0.5% methylcellulose (MC) (Shin-Etsu Chemical, Tokyo, Japan). Blood samples collected from the orbital venous plexus using capillary tubes 24 h after the last administration were mixed with heparin sodium (20 U/ml) (Ajinomoto Pharmaceuticals, Tokyo, Japan) and K_2_EDTA (2 mg/ml) (Wako Pure Chemical Industries). The numbers of lymphocytes in blood samples were determined using a Sysmex XT-2000*i* Automated Hematology Analyzer (Sysmex Corporation, Kobe, Japan).

### Induction of EAE

To induce EAE in Lewis rats, 0.5 mg/ml guinea pig myelin basic protein (MBP; Bachem AG, Bubendorf, Switzerland) dissolved in phosphate-buffered saline (PBS) solution was emulsified with an equal volume of Freund's complete adjuvant containing *Mycobacterium tuberculosis* H37Ra (Difco Laboratories, Detroit, MI, USA). Female Lewis rats were immunized by subcutaneous injection of guinea pig MBP emulsion (100 µg/rat) in the hind footpads under inhaled isoflurane anesthesia (Mylan Seiyaku, Tokyo, Japan). Rats immunized subcutaneously with the emulsion without MBP served as normal controls. Animals were examined daily for clinical signs of neurological deficits that were scored on a scale of 0 to 5 as follows: 0, no abnormality; 1, flaccid tail; 2, paralysis of one hind limb; 3, paralysis of both hind limbs; 4, paralysis of hind and forelimbs or involuntary urination; 5, death. EAE was induced in SJL/J mice using (Ser140)-myelin proteolipid protein (139–151) (PLP139-151; Bachem AG). PLP139-151 (500 µM) in PBS was emulsified with an equal volume of Freund's complete adjuvant containing *Mycobacterium tuberculosis* H37Ra. Female SJL/J mice were immunized by subcutaneous injection into the flank regions with emulsified PLP139-151 (50 nmol/mouse). Pertussis toxin (List Biological Laboratories, Campbell, CA, USA) dissolved in PBS was injected intravenously (100 ng/mouse/day) on the day of immunization and 2 days later. Mice immunized by subcutaneous injection of emulsion without PLP139-151 served as normal controls. Individual animals were examined daily for neurological deficits scored on a 0 to 6 scale as follows: 0, no abnormality; 1, flaccid tail; 2, hind limb weakness; 3, paralysis of one hind limb; 4, paralysis of bilateral hind limbs; 5, paralysis of hind and forelimbs or involuntary urination or moribund; 6, death. Food pellets were placed inside the cages for easy access to food and saline was administered subcutaneously when the animals had paralysis. No animals reached clinical score 5 (for rats) or 6 (for mice) or had to be euthanized during the study. At the end of the study, all animals were euthanized by inhalation of CO_2_.

### Measurement of Heart Rate and Mean Blood Pressure in rats

Male Lewis rats were randomized by weight to each experimental group. Polyethylene catheters were implanted into the femoral artery and vein of rats transiently anesthetized with inhaled isoflurane (Mylan Seiyaku). ASP4058 and fingolimod-P were dissolved in 10% DMSO (v/v) and 10 mM HCl in saline, or in 10% DMSO in saline, respectively. Systolic blood pressure (SBP) and diastolic blood pressure (DBP) were measured using a pressure amplifier (AP-601G, Nihon Kohden, Tokyo, Japan) connected to a pressure transducer (DX-360, Nihon Kohden), and heart rate was measured using a tachometer (AT-601G, Nihon Kohden) triggered by the arterial pulse wave. Following a stabilization period, respective vehicles, ASP4058, or fingolimod phosphate were administered by continuous intravenous infusion through a catheter inserted into the femoral vein for 10 min at a flow rate of 1 ml/kg/min using an infusion pump (KDS100, Neuroscience Inc, Tokyo, Japan). Heart rates and blood pressures were recorded for 20 min from the start of infusion. The mean blood pressure was calculated as follows: Mean blood pressure  =  (SBP – DBP)/3 + DBP.

### Analysis of Bronchoconstriction

Tracheotomy was performed on male Lewis rats anesthetized with pentobarbital sodium solution (Kyoritsu Seiyaku Corporation, Tokyo, Japan). A polyethylene catheter was inserted into a femoral vein for administration of compounds. Rats were mechanically ventilated using a small animal ventilator (Harvard Model 683, Harvard Apparatus, MA, USA) set at a stroke volume of approximately 2 ml and a rate of 90 breaths/min with positive end-expiratory pressure to prevent alveolar collapse. A cross-connector was attached to the respirator expiratory tubing to measure changes in airway pressure using a pressure transducer (TP-603T, Nihon Kohden) connected to an amplifier (AR-601G, Nihon Kohden). Pancuronium bromide (0.2 mg/kg) (MSD, Tokyo, Japan) was administered intravenously to attenuate spontaneous respiration and obtain a stable baseline. ASP4058 and fingolimod-P were dissolved in 10% DMSO and 10 mM HCl in saline or 10% DMSO in saline, respectively. After acquiring baseline airway pressures (baseline), the respective vehicles, ASP4058 or fingolimod-P was administered via continuous intravenous infusion through a catheter inserted into the femoral vein at a flow rate of 1 ml/kg/min using an infusion pump (KDS100, Neuroscience Inc).

### Plasma or Blood Concentrations of ASP4058 and Fingolimod Phosphate

Blood samples were collected from Lewis rats that received once-daily oral doses of ASP4058 or fingolimod for 14 days and from Lewis rats that received continuous intravenous infusion of ASP4058 or fingolimod-P, using the same method described above for measuring heart rate. Whole blood or plasma (separated by centrifugation) was stored at −20°C. Samples were prepared using protein precipitation, and analyses were determined using high-performance liquid chromatography-tandem mass spectrometry (API2000; AB SCIEX, MA, USA).

### Brain Distribution of ASP4058

Sprague Dawley rats (three rats per group) that received a single oral dose of [^14^C]ASP4058 (1 mg/kg as the free base) were anesthetized with inhaled isoflurane. Blood samples were collected from the abdominal aorta using a heparinized syringe 1, 4, 24, 72, and 168 h after administration and centrifuged to separate the plasma. An aliquot (100 µl) of plasma was dissolved in 2 ml of the tissue solubilizer Soluene-350 (PerkinElmer). The rats were then sacrificed by exsanguination and the whole brain was excised, weighed, and homogenized in saline. Approximately 500 mg of brain homogenate was weighed and solubilized with 2 ml of Soluene-350 with heating. Each sample was mixed with 10 ml of Hionic-Fluor scintillation fluid (PerkinElmer), and the radioactivity was measured using a liquid scintillation counter (2700TR, 1900CA, PerkinElmer). The radioactivity concentration, expressed as equivalents of ASP4058, was calculated as follows:
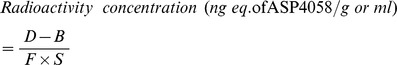



D: Radioactivity in the sample (dpm)

B: Background (dpm)

F: Specific radioactivity of the dosing solution (dpm/ng)

S: Amount of assay sample (g or ml)

### Statistical Analysis

GTPγS binding assays were performed in duplicate or triplicate, and the data were analyzed using SAS software (SAS Institute Inc., Cary, NC, USA). Concentration response curves were calculated using nonlinear regression analysis to fit a sigmoid maximal effect model using SAS software (SAS Institute), and concentrations yielding half-maximal effects (EC_50_) were determined from the curve. The EC_50_ values of each drug are represented as the geometric mean and 95% confidence interval (CI). All data acquired *in vivo* are expressed as the arithmetic mean ± standard error (S.E.). The significance of changes in peripheral lymphocyte counts were analyzed by comparing the vehicle control and treated groups using Dunnett's multiple comparison test and the 50% effective dose (ED_50_) values were calculated using the linear regression method with the lymphocyte number in the vehicle-treated group defined as 100%. Cumulative clinical scores of EAE model animals were analyzed by comparing the vehicle control and treated groups using Dunnett's multiple comparison test, and the differences in maximum clinical scores in EAE model animals between groups were analyzed using Steel's multiple comparison test. The differences in airway responses between control and ASP4058-treated or fingolimod-P-treated rats were analyzed using Student's *t* test or Dunnett's multiple comparison test, respectively.

## Results

### Effects of ASP4058 and fingolimod phosphate on S1P receptor subtypes

The agonistic effects of ASP4058 and fingolimod-P on human S1P receptor subtypes were evaluated using GTPγS binding assays. Low nanomolar concentrations of ASP4058 stimulated S1P_1_ and S1P_5_, and more than 100-times higher concentration was required to stimulate S1P_2_, S1P_3_, and S1P_4_. In contrast, low nanomolar concentrations of fingolimod-P stimulated S1P_1_ and S1P_5_ as well as S1P_3_ and S1P_4_ (Table 1). To compare human and rat S1P receptors, the agonistic effects of ASP4058 and fingolimod-P on rS1P_1_ and rS1P_3_ were further evaluated, and EC_50_ values of ASP4058 and fingolimod-P for rS1P_1_ and rS1P_3_ were found to be comparable to those for the cognate human receptors (Table 2). Further, receptor-binding screens were conducted to determine the affinity of ASP4058 for a wide range of receptors, ion channels and transporters, and to determine the target specificity of ASP4058. We found that 10 µM ASP4058 did not detectably inhibit ligand binding to any target by more than 30% (Table S1), indicating that ASP4058 is a specific agonist of S1P receptors.

**Table 1:** Agonistic effects of ASP4058 and fingolimod phosphate on human S1P receptor subtypes.

	EC_50_ (nM)				
	(95% CI)				
Compound	*hS1P_1_*	*hS1P_2_*	*hS1P_3_*	*hS1P_4_*	*hS1P_5_*
ASP4058	7.4	n.d.	920	2300	7.5
	(4.1–13)		(750–1100)	(1400–3900)	(5.3–11)
fingolimod-P	1.4	n.d.	2.9	2.2	0.86
	(0.58–3.5)		(2.0–4.1)	(1.9–2.7)	(0.76–0.98)

EC_50_ values were determined from concentration-response curves and are represented as the geometric mean with the 95% confidence interval (CI) from four independent experiments. fingolimod-P, fingolimod-phosphate; hS1P_1–5_, human S1P_1–5_.

**Table 2:** Agonistic effects of ASP4058 and fingolimod phosphate on rS1P_1_ and rS1P_3_.

	EC_50_ (nM) (95% CI)	
Compound	*rS1P_1_*	*rS1P_3_*
ASP4058	9.7 (2.4–40)	300 (120–730)
fingolimod-P	1.1 (0.83–1.6)	1.1 (0.69–1.6)

EC_50_ values were determined from concentration-response curves and are represented as the geometric mean and 95% confidence interval (CI) from three separate experiments using ASP4058 and four independent experiments using fingolimod phosphate. fingolimod-P, fingolimod-phosphate; rS1P_1,3_, rat S1P_1,3_.

### Effect of S1P receptor agonists on number of lymphocytes in peripheral blood

**Figure 2:**
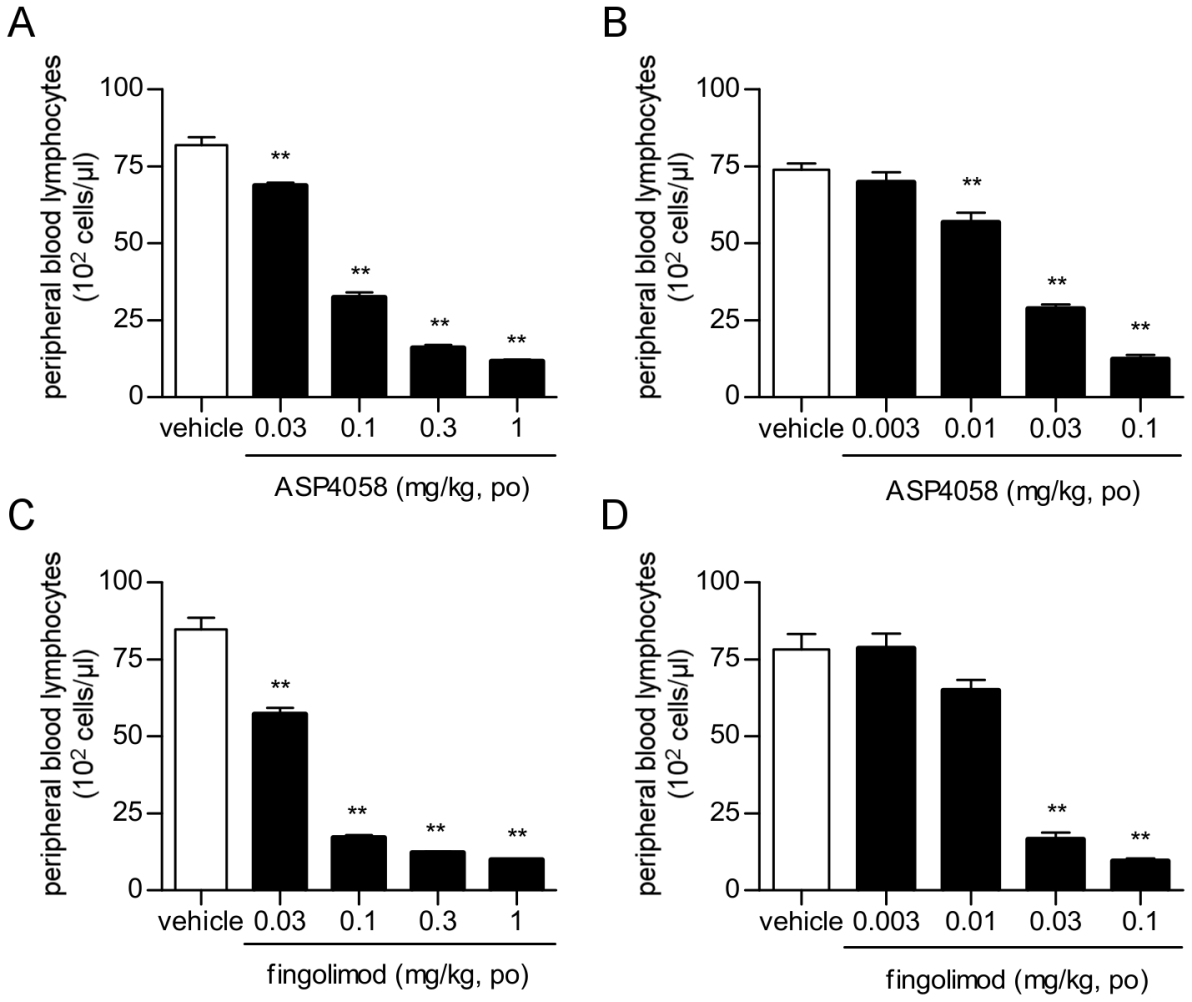
Effects of ASP4058 and fingolimod on the number of peripheral lymphocytes in Lewis rats. (A, C) The figure shows peripheral lymphocyte counts in blood samples taken 24 hours after single oral dose of ASP4058 (A) and fingolimod (C). (B, D) ASP4058 or fingolimod were administered to Lewis rats once daily for 21 days. The figure shows peripheral lymphocyte counts in blood samples taken 24 hours after the last administration of ASP4058 (B) and fingolimod (D). All data represent the mean ± S.E. (*n* = 5). **P<0.01 compared with the vehicle-treated group (Dunnett's multiple comparison test).

We next determined the effect of S1P receptor agonists on peripheral lymphocyte counts. A single oral dose of ASP4058 reduced the peripheral lymphocyte counts of Lewis rats as a function of dose (ED_50_ = 0.10 mg/kg 24 h after treatment) (Fig. 2A). Repeated dosing of ASP4058 for 21 days led to a more potent reduction in the number of peripheral lymphocytes (ED_50_ = 0.023 mg/kg 24 hours after the last dose) (Fig. 2B). Similarly, fingolimod reduced peripheral lymphocyte counts with ED_50_ values of 0.041 or 0.020 mg/kg 24 h after single or 21-day repeated oral treatments, respectively (Fig. 2C, 2D).

### Plasma or blood concentrations of S1P receptor agonists

**Figure 3:**
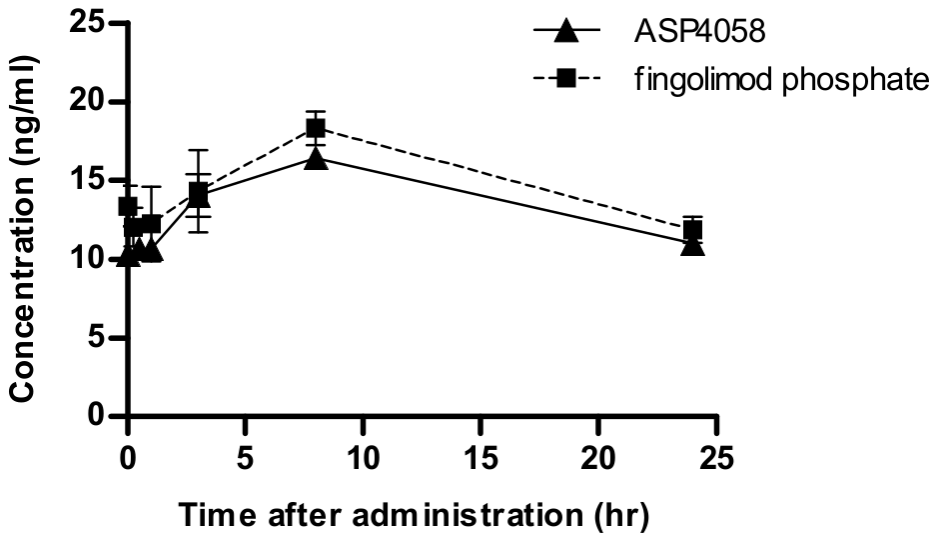
Plasma or blood concentrations of ASP4058 and fingolimod-P after repeated dosing. ASP4058 (0.1 mg/kg) or fingolimod (0.1 mg/kg) was administered once-daily for 14 days in male Lewis rats. Plasma concentration of ASP4058 and blood concentration of fingolimod phosphate (fingolimod-P) in rats were measured just before the last administration, 0.25 (for fingolimod-P) or 0.5 (for ASP4058), 1, 3, 8, and 24 h after the last administration. Data represent the mean ± S.E. (*n* = 5).

We determined the plasma concentrations of ASP4058 and the blood concentration of fingolimod-P in Lewis rats after 14 days of repeated administration to investigate their pharmacokinetics at the dose that induced the maximal effect on lymphocyte counts (0.1 mg/kg each of ASP4058 or fingolimod) (Fig. 3). The maximum plasma concentrations of ASP4058 and the maximum blood concentrations of fingolimod-P under these conditions were 16.4±0.463 and 18.6±1.14 ng/ml, respectively. Further, the distribution of ASP4058 in the brain was investigated by administering [^14^C]ASP4058 to Sprague Dawley rats. The radioactivity concentrations in the plasma and brain at 24 h after a single oral administration of [^14^C]ASP4058 (1 mg/kg) were 129±13.6 ng eq. of ASP4058/ml and 315±8.29 ng eq. of ASP4058/g, respectively. The radioactivity concentrations in the brain decreased almost in parallel with those in plasma, and the brain-to-plasma concentration ratio remained within the range of 2.4–2.9 from 4 h to 168 h after administration.

### Effects of S1P receptor agonists on acute monophasic EAE in rats

**Figure 4:**
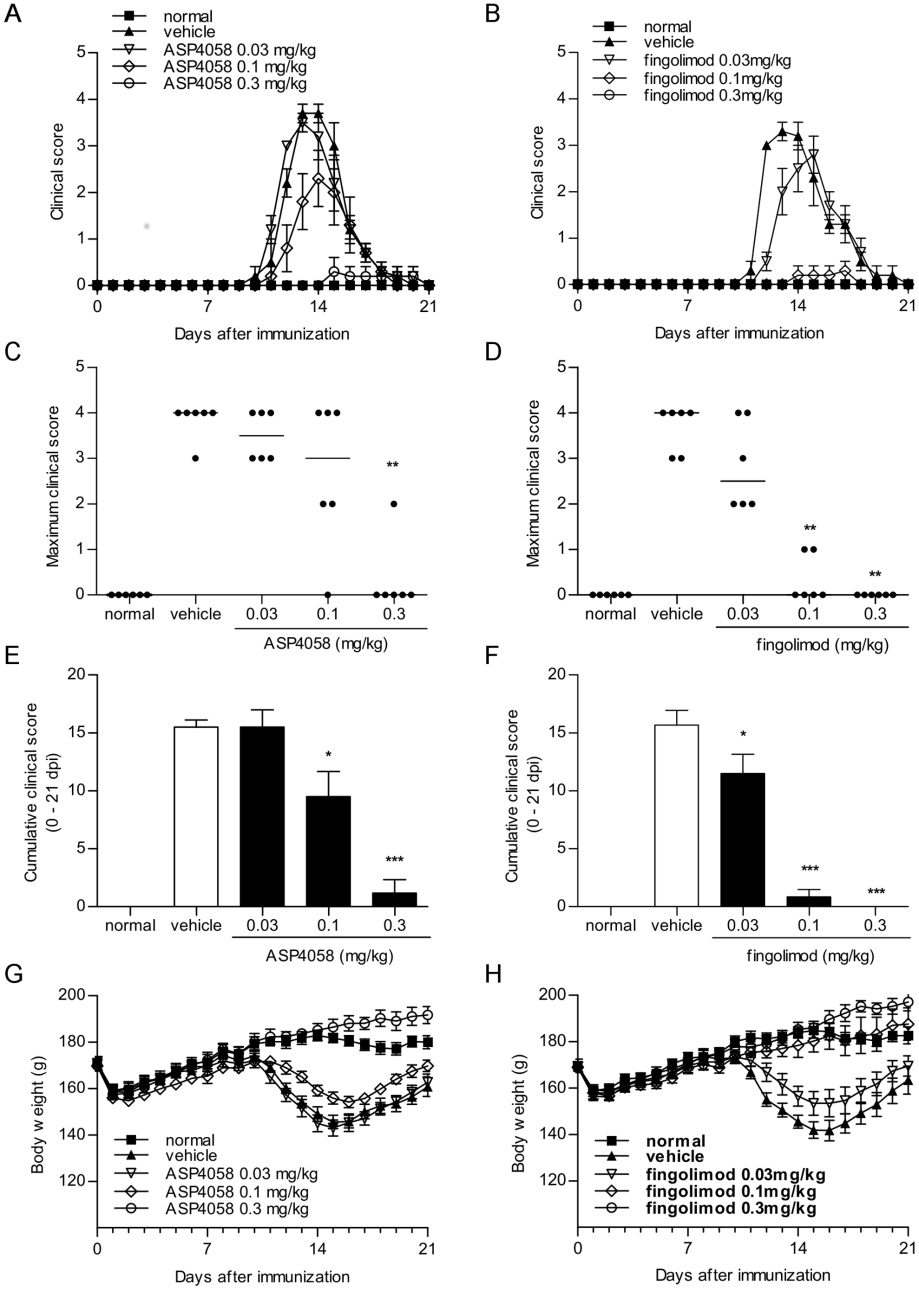
Prophylactic effect of ASP4058 and fingolimod on acute monophasic EAE in rats. Experimental autoimmune encephalomyelitis (EAE) was induced in female Lewis rats, and each animal was examined daily for neurological deficits. Rats were randomized by weight and orally administered 0.5% methylcellulose (MC) (vehicle), ASP4058 (0.03, 0.1, 0.3 mg/kg) or fingolimod (0.03, 0.1, 0.3 mg/kg) once daily for 21 days from the day of immunization. (A, B) Time course of EAE development in Lewis rats. (C, D) Maximum clinical scores of individual animals. Bars indicate the median value of each group. **P<0.01 compared with vehicle-treated group (Steel's multiple comparison test). (E, F) The cumulative clinical score for each animal was calculated by adding daily clinical scores during the experimental period (0–21 dpi). *P<0.05, ***P<0.001 compared with vehicle-treated group (Dunnett's multiple comparison test). (G, H) Effects of ASP4058 and fingolimod on the decrease of body weight in EAE rats. All data represent the mean ± S.E. (*n* = 6).

All vehicle-treated rats immunized with MBP developed typical clinical symptoms of EAE lasting from 10 to 21 days postimmunization (dpi) (Fig. 4A, 4B). ASP4058 (0.03, 0.1 or 0.3 mg/kg) or fingolimod (0.03, 0.1 or 0.3 mg/kg) was administered from the day of immunization to evaluate their prophylactic effects. ASP4058 reduced the clinical score in a dose-dependent manner and the cumulative clinical score from day 0 to 21 dpi at 0.03, 0.1 and 0.3 mg/kg were 15.5±1.48, 9.50±2.17 and 1.17±1.17, respectively, while that of vehicle-treated group was 15.5±0.619 (Fig. 4E). A significant change in the maximal clinical score was observed on administration of 0.3 mg/kg (Fig. 4C). Further, ASP4058 prevented decreases in body weight of EAE rats (Fig. 4G). Similar effects were noted with fingolimod treatment (Fig. 4). While the cumulative clinical score from day 0 to 21 dpi among vehicle-treated group was 15.7±1.28, the cumulative clinical scores (day 0–21 dpi) among groups treated with 0.03, 0.1, and 0.3 mg/kg fingolimod were 11.5±1.65, 0.83±0.65, and 0.0±0.0, respectively (Fig. 4F). Significant change in the maximal clinical score was observed on administration of fingolimod at doses ≥0.1 mg/kg (Fig. 4D).

### Effect of S1P receptor agonists on relapsing-remitting EAE in mice

**Figure 5:**
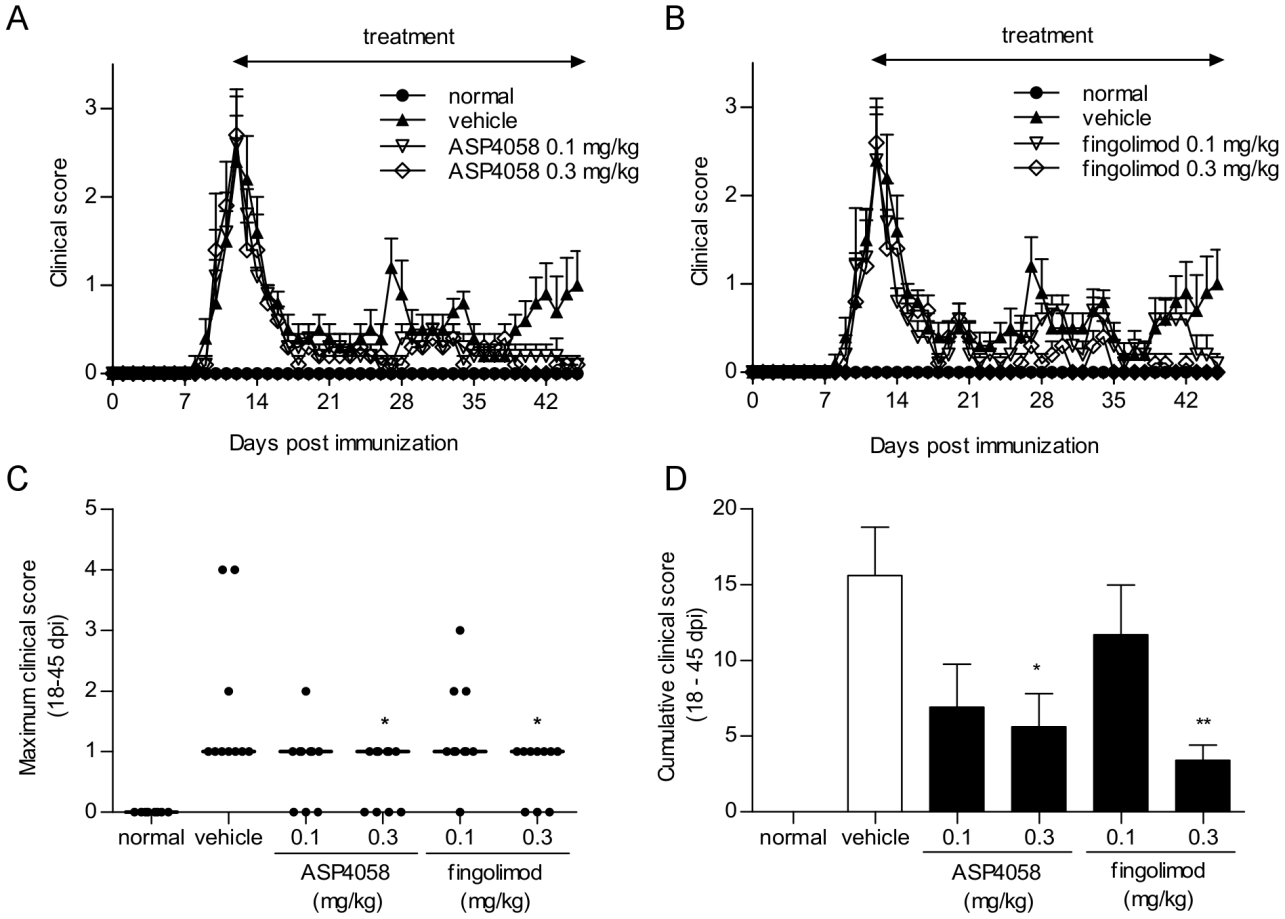
Prophylactic effect of ASP4058 and fingolimod on relapsing-remitting EAE in mice. Experimental autoimmune encephalomyelitis (EAE) was induced in female SJL/J mice, and each animal was examined daily for neurological deficits. Mice were randomized by weight and maximum clinical scores during 0–12 dpi and orally administered 0.5% methylcellulose (MC) (vehicle), or 0.1 and 0.3 mg/kg each of ASP4058 or fingolimod once daily from 12 dpi to the end of experiment (45 dpi). (A, B) Time course of EAE development in mice treated with ASP4058 (A) or fingolimod (B). The treatment period is indicated by an arrow. Clinical scores represent the mean ± S.E. (*n* = 10). (C) Maximum clinical scores of individual animal during the relapse-remitting phase (18–45 dpi). Bars indicate the median value of each group. *P<0.05 compared with the vehicle treated group (Steel's multiple comparison test). (D) Cumulative clinical scores during the relapse-remitting phase (18–45 dpi) were calculated. The results represent the mean ± S.E. (*n* = 10). *P<0.05, **P<0.01 compared with the vehicle-treated group (Dunnett's multiple comparison test).

We examined whether ASP4058 and fingolimod prevent the relapse of EAE using a mouse model of relapsing-remitting EAE. SJL mice immunized with PLP139-151 and boosted with pertussis toxin developed relapsing-remitting EAE. The acute phase appeared around 8 dpi, reached the maximum score at 12 dpi, and remitted by 18 dpi (Fig. 5A, 5B). Once-daily administration of vehicle, 0.1 and 0.3 mg/kg each of ASP4058, or fingolimod was started from the peak of acute phase (12 dpi), and treatment was repeated until 45 dpi. While several relapses occurred among mice in the vehicle-treated group after remission of acute clinical symptoms, and the cumulative clinical score during the relapse-remitting phase (18–45 dpi) was 15.6±3.18, administration of ASP4058 maintained the clinical score at a relatively low level, and the cumulative clinical scores (18–45 dpi) among the groups treated with 0.1 and 0.3 mg/kg ASP4058 were 6.90±2.85 and 5.60±2.21, respectively (Fig. 5D). Similarly, fingolimod suppressed the clinical symptoms as a function of dose, and the cumulative clinical score among the groups treated with 0.1 and 0.3 mg/kg were 11.7±3.28 and 3.40±1.01, respectively (Fig. 5D). Further, 0.3 mg/kg of ASP4058 or fingolimod significantly reduced the maximal clinical score (Fig. 5C). The number of peripheral lymphocytes was determined 24 h after the last dose, and the ED_50_ values for ASP4058 and fingolimod were 0.063 and 0.060 mg/kg, respectively.

### Cardiovascular effects of S1P receptor agonists

**Figure 6:**
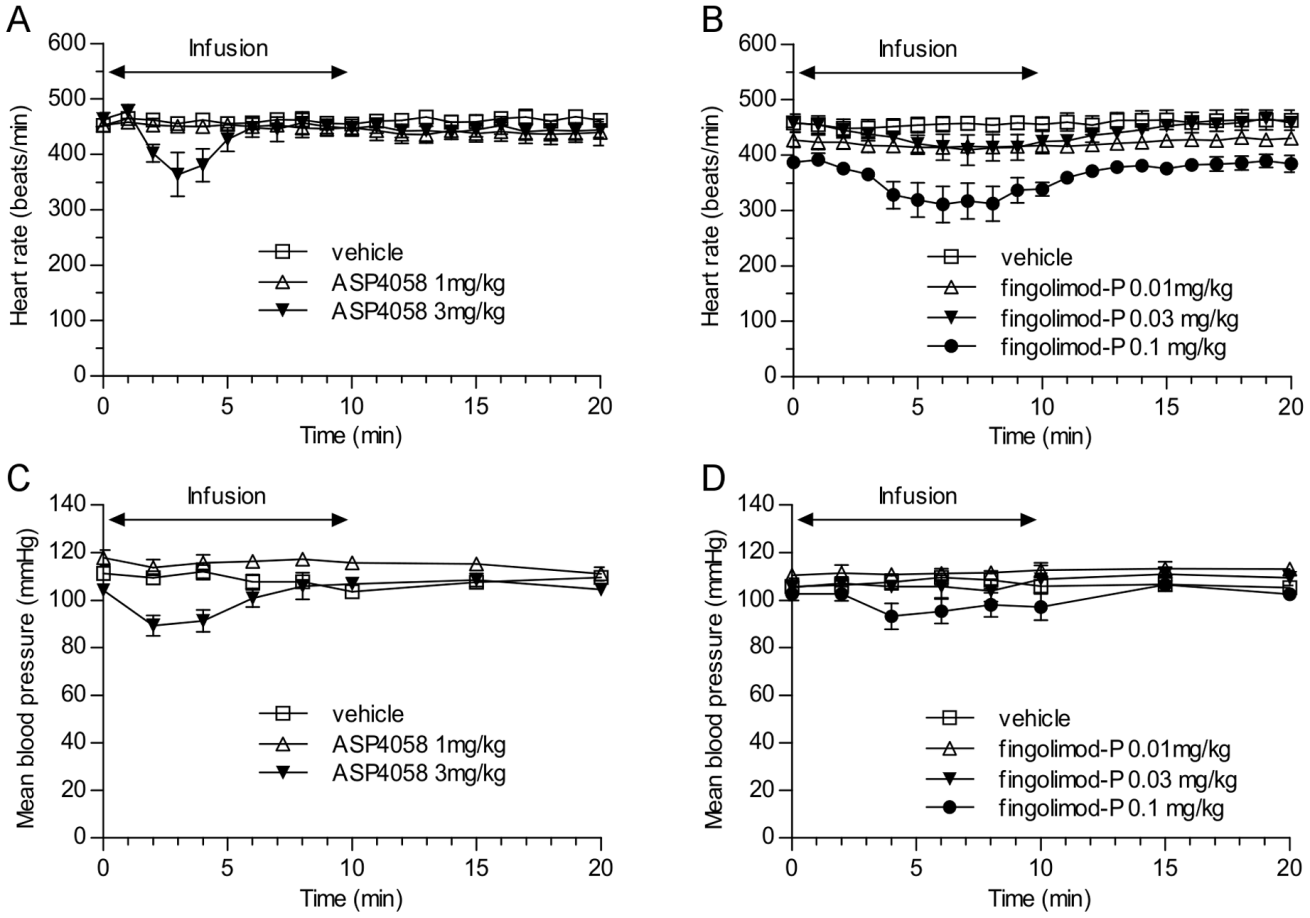
Effects of ASP4058 and fingolimod-P on heart rate and mean blood pressure in conscious rats. Vehicle, ASP4058 (1 or 3 mg/kg) or fingolimod phosphate (fingolimod-P) (0.01, 0.03, or 0.1 mg/kg) were continuously administered for 10 min using an infusion pump (1 ml/kg/min) through a catheter inserted into the femoral vein, and the effects of ASP4058 and fingolimod-P on heart rate and mean blood pressure were determined (ASP4058, A and C, respectively; fingolimod-P, B and D, respectively). Heart rates and mean blood pressures were recorded for 20 min after the start of infusion. Heart rate was determined at 1-min intervals, and mean blood pressures were determined 0, 2, 4, 6, 8, 10, 15, and 20 min after the start of infusion. All values represent the mean ± S.E. for 5 rats per group, except for the 20-min time points of the groups treated with 1 mg/kg of ASP4058 (mean ± S.E. for 4 rats) or 0.1 mg/kg fingolimod phosphate (mean ± S.E. for 3 rats).

We next determined the effects of ASP4058 or fingolimod-P on heart rate and mean blood pressure in conscious rats. ASP4058 (1, 3 mg/kg) and fingolimod-P (0.01, 0.03 or 0.1 mg/kg) were administered via continuous intravenous infusion for 10 min, and heart rates and blood pressures were recorded for 20 min. Although the administration of ASP4058 at 1 mg/kg did not cause a marked decrease in heart rate, ASP4058 administered at 3 mg/kg transiently reduced heart rate, which returned to the baseline level during the infusion period (Fig. 6A). The mean blood pressure of rats treated with the higher dose simultaneously (but only transiently) decreased during the infusion (Fig. 6C). Administration of 0.01 mg/kg of fingolimod-P did not show any obvious effect on heart rate, however, treatment with 0.03 mg/kg of fingolimod-P induced a slight decrease, and 0.1 mg/kg induced a marked reduction in heart rate, which returned to the basal level after the infusion was terminated (Fig. 6B). Further, a simultaneous decrease in the mean blood pressure of rats treated with 0.1 mg/kg was also noted (Fig. 6D).

The plasma or blood concentrations during and after the infusion of ASP4058 (1 mg/kg) and fingolimod-P (0.03 mg/kg) were evaluated in separate groups (Table 3). The maximum plasma concentrations of ASP4058 and the maximum blood concentration of fingolimod-P were 483±18.0 ng/ml and 18.49±1.38 ng/ml, respectively (Table 3).

**Table 3:** Plasma concentrations of ASP4058 and blood concentration of fingolimod phosphate during and after intravenous infusion of rats.

		Plasma or Blood Concentration (ng/ml)			
Compound	Dose (mg/kg)	2 min	5 min	10 min	20 min
ASP4058	1	317±3.61	448±18.7	483±18.0	200±7.42
fingolimod-P	0.03	9.68±0.95	14.17±1.27	18.49±1.38	3.04±0.60

All values represent the mean ± S.E. for three rats per group. fingolimod-P, fingolimod-phosphate.

### Effects of S1P receptor agonists on bronchoconstriction

**Figure 7:**
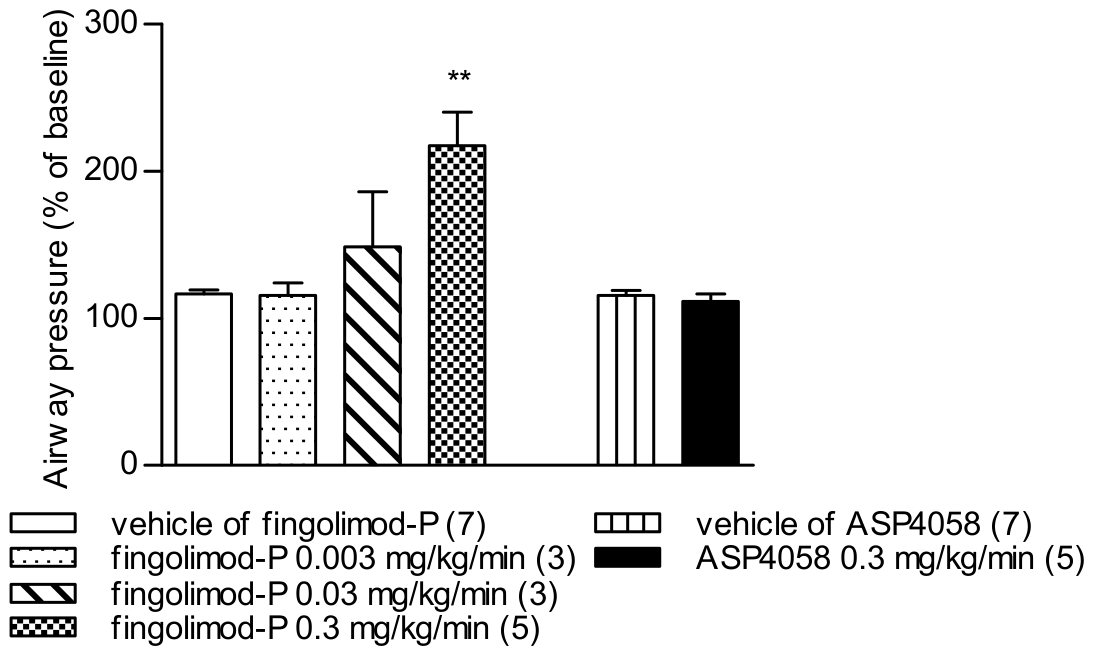
Effects of ASP4058 or fingolimod phosphate on bronchoconstriction in anesthetized rats. To assess the effect of compound on base-line airway pressure, ASP4058 (0.3 mg/kg/min) or fingolimod phosphate (fingolimod-P) (0.003, 0.03, 0.3 mg/kg/min) were administered by continuous intravenous infusion, and airway pressure was measured just before the initiation of compound infusion (baseline) and approximately 20 min after the initiation of compound infusion, which is sufficient time to reach a plateau. Results are shown as the percent change from baseline value and represent the mean ± S.E. The number of animals in each group is shown in parentheses. **P<0.01 compared with the vehicle-treated group (Dunnett's multiple comparison test).

To determine whether or not S1P receptor agonists affect pulmonary function, ASP4058 or fingolimod-P was administered to anesthetized rats using continuous intravenous infusion. Airway resistance began to increase approximately 5 min after the beginning of fingolimod-P infusion and reached a plateau at approximately 10 min after start of infusion. A statistically significant increase in airway resistance was induced by infusion of 0.3 mg/kg/min fingolimod-P, while the effect of infusing 0.3 mg/kg/min ASP4058 was similar to that of the vehicle (Fig. 7).

## Discussion

Here we determined the preclinical profiles of ASP4058, a novel S1P_1_, S1P_5_ agonist synthesized by Astellas Pharma Inc. ASP4058 was effective in rodent EAE models and showed favorable safety profiles based on the results of tests for bradycardia and bronchoconstriction when compared with fingolimod. Thus, ASP4058 may provide a novel therapeutic option for patients with MS that is safer than nonselective S1P receptor agonists such as fingolimod.

S1P_1_ is a key mediator of the immunomodulatory effects of S1P receptor agonists [17], [19]. S1P receptor agonists exert these effects, at least in part, by inducing long-term downregulation of S1P_1_ expressed by lymphocytes, which causes the sequestration of cells in lymphoid tissues and prevents their migration to target organs [11]. Consistent with these findings, treatment of rats with ASP4058 reduced the number of peripheral lymphocytes. The ED_50_ of ASP4058 required to reduce the number of peripheral lymphocytes after a single dose was higher by a factor of 2.4 than that of fingolimod; however, the effective dose of both compounds after repeated administration for 21 days were equivalent, suggesting a cumulative effect of ASP4058 on reducing peripheral lymphocytes. Trafficking of T cells and B cells depends on S1P_1_, whereas the trafficking of natural killer (NK) cells requires expression of S1P_5_ [20], [21]. Because ASP4058 is an agonist of S1P_1_ and S1P_5_, ASP4058 may affect not only T and B cells but NK cells as well. Further investigation is required to identify the lymphocyte subsets affected by ASP4058.

Next, we determined the effect of ASP4058 and fingolimod in rodent EAE, which is an autoimmune disease mediated by lymphocytes [22]. While both ASP4058 and fingolimod exerted a prophylactic effect on EAE in Lewis rats, the dose of ASP4058 required to achieve the maximum effect compared with fingolimod was higher by a factor of approximately 3 in this model, possibly due to difference in dose required for each compound to reduce the numbers of lymphocytes at the beginning of the treatment. Lewis rats serve as an acute model of EAE in which clinical symptoms appear approximately 10 days after immunization and remit shortly after onset. Therefore, the immunomodulatory effects of S1P receptor agonists in the early phase may contribute significantly to the efficacy in EAE model in Lewis rats. Consistent with this hypothesis, the maximum effective dose of each compound in this model was equal to the dose required to maximally reduce the population of peripheral lymphocytes after a single administration.

We further investigated the effects of ASP4058 and fingolimod on SJL/J mice with EAE, which display a relapsing-remitting clinical course. Each compound was administered after clinical symptoms appeared to investigate the effects on relapse. ASP4058 and fingolimod significantly reduced clinical symptoms during relapse at the same dose (0.3 mg/kg), which may be attributed to the equivalent potency of both compounds for reducing lymphocyte numbers in this system. The results acquired using both EAE models suggest that ASP4058 ameliorates EAE primarily by reducing the number of peripheral lymphocytes, which prevents infiltration of encephalitogenic lymphocytes into the CNS.

In addition to their immunologic function, evidence indicates that S1P receptor agonists directly affect the CNS. S1P receptors are expressed by oligodendrocytes, astrocytes, microglia, and neurons [12]. EAE is attenuated and fingolimod efficacy is lost in conditional null-mutant mice lacking S1P_1_ by astrocytes [23], which suggests that S1P receptor agonists may mitigate EAE through functional antagonism of S1P_1_ expressed by astrocytes. Further, other studies indicate the function of S1P receptors expressed by cells of the oligodendrocyte lineage. Oligodendrocytes are myelinating cells of the CNS, which are the principal target of immune attack in MS. Loss of myelin and the failure of remyelination by oligodendrocytes contribute to the functional impairment of patients with MS [24]. Fingolimod-P treatment rescues oligodendrocyte progenitor cells from undergoing apoptosis in a death-inducing environment through S1P_1_ signaling [25]. S1P or fingolimod-P promotes the survival of mature oligodendrocytes through S1P_5_ [26], [27]. Fingolimod-P enhances remyelination in lysolecithin-induced demyelination in cerebellar slice culture, and an agonist specific for S1P_5_ shows a trend toward an increase in remyelination [28]. ASP4058 acts agonistically on S1P_1_ and S1P_5_ and penetrates the CNS. Thus, the beneficial effects of ASP4058 for treating EAE may involve its direct effect on cells of the CNS.

In clinical trials, fingolimod induced adverse events such as reduction of heart rate or mean FEV1 [29]. Preclinical findings that bradycardia induced by a nonselective S1P receptor agonist administered to wild-type mice is abolished in S1P_3_ knockout mice and that an S1P_1_-selective agonist does not induce bradycardia suggest that S1P_3_ signaling induces bradycardia [17], [30]. Consistent with these reports, ASP4058, which has weak agonistic activity for S1P_3_, required a higher dose than fingolimod-P to induce bradycardia in conscious rats. The safety margin between the lymphocyte-reducing effect and the bradycardia-inducing effect was evaluated by determining the plasma or blood concentrations of compounds. The concentrations of fingolimod-P required to change heart rate and to exert the maximal effect on lymphocyte counts in rats were similar. In contrast, the concentration of ASP4058 required to induce bradycardia was more than 30-times higher than that required to induce lymphopenia. These results suggest that ASP4058 presents less risk for adverse cardiovascular events than fingolimod-P because of its low activity for S1P_3_. Transient bradycardia was observed in healthy humans treated with the S1P_1_, S1P_5_ agonist BAF312, which suggest species-specific differences in S1P receptor specificity for first-dose cardiac effects [31]. Therefore, whether or not ASP4058 exerts cardiovascular effects in human remains to be determined.

S1P affects airway constriction in a Rho-dependent manner. For example, S1P stimulates contraction of human airway smooth muscle cells and guinea pig tracheal strips [32], [33] and enhances methacholine-induced contraction of guinea pig trachea [32]. Further, systemic administration of S1P to mice increases cholinergic reactivity of isolated bronchi and lungs [34]. In addition, fingolimod, but not the selective S1P_1_ agonist AUY954, induces bronchoconstriction or airway hyperreactivity, and hyperresponsiveness to 5-hydroxytryptamine induced by fingolimod-P is not observed in trachea isolated from S1P_3_-deficient mice [18]. These data suggest that the airway response induced by the S1P signal is mediated by S1P_3_. Consistent with these reports, in the present study, continuous injection of fingolimod-P significantly increased bronchoconstriction, but the same dose of ASP4058 did not. The exact mechanism responsible for the pulmonary adverse event observed in the clinical trial of fingolimod is unknown; however, the effect of fingolimod on the airway response might be involved to some extent. Therefore, our data suggest that ASP4058 treatment presents less risk for adverse pulmonary events than nonselective S1P receptor agonists such as fingolimod.

In summary, we show here that ASP4058 is a selective agonist of S1P_1_ and S1P_5_, which effectively treats rodents with EAE. Further, ASP4058 exhibited a wide safety margin for bradycardia and bronchoconstriction. We therefore consider ASP4058 a potential new therapeutic option for the treatment of patients with MS, which is safer than nonselective S1P receptor agonists such as fingolimod. ASP4058 may also be useful in treating other autoimmune diseases given its ability to significantly reduce the population of peripheral lymphocytes.

## Supporting Information

Table S1 **Inhibitory effect of ASP4058 on binding of radioligands to various receptors, ion channels and transporters.** Receptor-binding screens were conducted by Sekisui Medical Co., Ltd (Tokyo, Japan) to determine the affinity of ASP4058 for various receptors, ion channels and transporters.
(PDF)

Checklist S1 **ARRIVE check list.**
(PDF)
