# Supplementary material for: ASP4058, a Novel Agonist for Sphingosine 1-Phosphate Receptors 1 and 5, Ameliorates Rodent Experimental Autoimmune Encephalomyelitis with a Favorable Safety Profile
Source: PLoS One. 2014 Oct 27;9(10):e110819. doi: 10.1371/journal.pone.0110819 (PMC4210206; doi:10.1371/journal.pone.0110819)
Supplement: Table S1 — Inhibitory effect of ASP4058 on binding of radioligands to various receptors, ion channels and transporters. Receptor-binding screens were conducted by Sekisui Medical Co., Ltd (Tokyo, Japan) to determine the affinity of ASP4058 for various receptors, ion channels and transporters. (PDF) [file pone.0110819.s001.pdf]

Table S1. Inhibitory effect of ASP4058 on binding of radioligands to various receptors, ion channels and transporters

Assay	Inhibition (%)		
	ASP4058	*Positive substance*	
Adenosine A1 (Rat)	1.81	100.00	(DPCPX)
α1-Adrenergic (Non-selective) (Rat)	0.00	100.00	(Prazosin)
α2-Adrenergic (Non-selective) (Rat)	0.00	100.00	(Yohimbine)
β-Adrenergic (Non-selective) (Rat)	0.00	100.00	((±)-Propranolol)
Angiotensin AT1 (Human)	6.15	99.63	(Angiotensin II)
Angiotensin AT2 (Mouse)	0.98	100.00	(Angiotensin II)
Bradykinin B2 (Human)	0.00	100.00	(HOE140)
Ca Channel (Type L, Dihydropyridine) (Rat)	1.49	99.28	(Nitrendipine)
Ca Channel (Type N) (Rat)	0.00	100.00	(ω-Conotoxin GVIA)
CCK A (Human)	1.46	100.00	(CCK-8)
CCK B (Human)	0.00	95.74	(CCK-8)
CRF1 (Human)	2.81	100.00	(Urocortin human)
Dopamine D1 (Rat)	4.27	100.00	(R(+)-SCH-23390)
Dopamine D2 Short (Human)	0.00	98.06	((+)-Butaclamol)
Dopamine Transporter (Human)	0.00	100.00	(GBR12909)
Estrogen (Rat)	1.57	93.71	(β-Estradiol)
Endothelin ETA (Human)	0.00	99.34	(Endothelin-1)
Endothelin ETB (Human)	10.38	100.00	(Endothelin-1)
GABA A (Agonist Site) (Rat)	0.62	100.00	(Muscimol)
GABA A (BZ Central) (Rat)	0.89	100.00	(Diazepam)
GABA B (Rat)	13.43	99.13	(GABA)
Glutamate (AMPA) (Rat)	2.82	99.65	((S)-AMPA)
Glutamate (Kainate) (Rat)	0.00	100.00	(Kainic acid)
Glutamate (NMDA Agonist Site) (Rat)	3.22	100.00	(L-Glutamic acid)
Glutamate (NMDA Glycine Site) (Rat)	16.00	96.66	(MDL105,519)
Glycine (Strychnine Sensitive) (Rat)	0.00	98.74	(Strychnine)
Histamine H1 (Central) (Guinea pig)	0.00	100.00	(Pyrilamine)
Histamine H2 (Rat)	0.00	96.46	(Cimetidine)
Histamine H3 (Rat)	0.51	100.00	((R)(−)-α-Methylhistamine)
K Channel KATP (Rat)	0.00	100.00	(Glibenclamide)
K Channel SkCa (Rat)	0.00	99.60	(Apamin)
Leukotriene B4 (Guinea pig)	0.00	96.69	(Leukotriene B_4)
Leukotriene D4 (Guinea pig)	0.00	100.00	(Leukotriene D_4)
Melatonin MT1 (Human)	28.47	100.00	(Melatonin)
Muscarinic (Non-selective) (Rat)	3.14	100.00	(Atropine)

Muscarinic M1 (Human)	3.45	100.00	(Atropine)
Muscarinic M2 (Human)	12.35	99.97	(Atropine)
Na Channel Site 2 (Rat)	0.00	100.00	(Dibucaine)
Neurokinin NK1 (Human)	7.45	100.00	(L-703,606)
Neurokinin NK2 (Human)	0.00	98.84	(Neurokinin A)
Neurokinin NK3 (Human)	0.00	99.50	(Senktide)
Norepinephrine Transporter (Human)	0.00	100.00	(Desipramine)
Nicotinic Ni (Rat)	4.68	96.17	((±)-Nicotine)
Opiate (Non-selective) (Rat)	0.00	91.57	(Naloxone)
Opiate μ (Human)	0.00	97.43	(DAMGO)
Oxytocin (Rat)	1.25	96.19	(Oxytocin)
PAF (Rabbit)	0.00	99.85	(PAF)
Serotonin 5HT1 (Non-selective) (Rat)	0.00	95.90	(Serotonin)
Serotonin 5HT2B (Human)	0.00	99.70	(Serotonin)
Serotonin Transporter (Human)	0.00	100.00	(Imipramine)
Sigma (Non-selective) (Guinea pig)	0.00	100.00	(Haloperidol)
Testosterone (Human)	1.91	98.69	(Testosterone)
Vasopressin V1 (Rat)	2.71	100.00	([Arg^8]-Vasopressin)
VIP 1 (Human)	0.00	99.65	(VIP)

Test substance concentration was 10 μM. Positive substance concentration was 1 μM for HOE140, urocortin human, endothelin-1, leukotriene B_4, leukotriene D_4, and VIP, or 10 μM for the others. All data represent the mean values of duplicate measurements. The inhibition rate was calculated as $[1 - (B - N) / (B_0 - N)] \times 100$ (%), where B is total bound radioactivity in the presence of the test substance (individual value), B_0 is total bound radioactivity in the absence of the test substance (mean value), and N is nonspecific bound radioactivity (mean value).
